# Effect of Indoxyl Sulfate on the Repair and Intactness of Intestinal Epithelial Cells: Role of Reactive Oxygen Species’ Release

**DOI:** 10.3390/ijms20092280

**Published:** 2019-05-08

**Authors:** Simona Adesso, Marco Ruocco, Shara Francesca Rapa, Fabrizio Dal Piaz, Biagio Raffaele Di Iorio, Ada Popolo, Giuseppina Autore, Fuyu Nishijima, Aldo Pinto, Stefania Marzocco

**Affiliations:** 1 Department of Pharmacy, University of Salerno, I-84084 Fisciano, Salerno, Italy; sadesso@unisa.it (S.A.); maruocco.1@gmail.com (M.R.); s.rapa@studenti.unisa.it (S.F.R.); apopolo@unisa.it (A.P.); autore@unisa.it (G.A.); pintoal@unisa.it (A.P.); 2 Department of Medicine, University of Salerno, I-84084 Fisciano, Salerno, Italy; fdalpiaz@unisa.it; 3 UOC of Nephrology, “A. Landolfi Hospital”, I-83029 Solofra, Avellino, Italy; br.diiorio@gmail.com; 4 Pharmaceuticals Division, Kureha Corporation, 169-8503 Tokyo, Japan; f-nishijima@kureha.co.jp

**Keywords:** indoxyl sulfate, intestinal epithelial cells, chronic kidney disease, oxidative stress, AST-120

## Abstract

Chronic kidney disease (CKD) is characterized by an oxidative stress status, driving some CKD-associated complications, even at the gastrointestinal level. Indoxyl Sulfate (IS) is a protein-bound uremic toxin, poorly eliminated by dialysis. This toxin is able to affect the intestinal system, but its molecular mechanism/s in intestinal epithelial cells (IECs) remain poorly understood. This study’s aim was to evaluate the effect of IS (31.2–250 µM) on oxidative stress in IEC-6 cells and on the intactness of IECs monolayers. Our results indicated that IS enhanced oxidative cell damage by inducing reactive oxygen species (ROS) release, reducing the antioxidant response and affecting Nuclear factor (erythroid-derived 2)-like 2 (Nrf2) nuclear translocation as well its related antioxidant enzymes. In the wound healing assay model, IS reduced IEC-6 migration, slightly impaired actin cytoskeleton rearrangement; this effect was associated with connexin 43 alteration. Moreover, we reported the effect of CKD patients’ sera in IEC-6 cells. Our results indicated that patient sera induced ROS release in IEC-6 cells directly related to IS sera content and this effect was reduced by AST-120 serum treatment. Results highlighted the effect of IS in inducing oxidative stress in IECs and in impairing the intactness of the IECs cell monolayer, thus significantly contributing to CKD-associated intestinal alterations.

## 1. Introduction

Chronic kidney disease (CKD) progression induces multiple organs dysfunctions with various clinical aspects contributing to uremic syndrome. In spite of pharmacological and dialytic treatments, these aspects remain present and combined with multiple complications during CKD [1,2,3]. CKD is ascribed to the progressive retention of a large number of compounds that are excreted by healthy kidneys. These retained compounds are called uremic toxins because of their damaging effects on various physiological functions in CKD patients [1,2].

The accumulation of uremic toxins, such as indoxyl sulfate (IS), seems to be involved in the progression of renal failure and in its associated complications. IS is a protein-bound uremic toxin, which, therefore, is poorly removed by dialysis. IS derives from dietary tryptophan’s metabolism and the intestine has a primary role in IS production: the intestinal microbiota metabolizes tryptophan into indole and after its intestinal absorption, indole is further converted by the liver into IS [4]. Several evidences indicate that serum IS levels are markedly enhanced in renal disease patients and the high levels of IS seem to be associated with CKD severity [5] and related to an increase of mortality in CKD patients [6]. IS is a nephro-vascular toxin that induces nephrotoxicity above all on tubular cells, inhibits endothelial cells proliferation, and is also capable of inducing free radicals [7]. It has been reported that IS also increases inflammation and oxidative stress in macrophages and, more recently, in central nervous system cells, thus contributing to immune dysfunctions and neurodegenerative complications often observed in these patients [8,9,10].

Intracellular free radicals, such as reactive oxygen species (ROS), play a fundamental role in signal transduction, both during normal conditions and in pathological diseases as CKD. However, ROS overproduction, not balanced by a proper anti-oxidant response, could be the first cause of DNA damage and protein dysfunction [11]. It has been reported that in CKD patients is often observed an enhanced oxidative stress status, also at the beginning of the disease, becoming more severe toward the end-stage of the renal disease [1,12]. Moreover, it has also been described that oxidative stress may induce significant modifications in healthy kidneys, similarly to those observed in CKD patients [13]. 

Oxidative stress is also involved in intestinal damage inflammation associated with different diseases. ROS and their oxidized products regulate transcription factors and redox-sensitive signaling pathways, which control inflammatory processes within the intestinal layer. The intestinal epithelium plays an important role by forming a physical and biochemical barrier to commensal and pathogenic microorganisms and by maintaining the immunoregulatory functions that influence the homeostasis of mucosal immune cells. Moreover, intestinal homeostasis is granted by the presence of a proteic structure, allowing inter and intracellular transport phenomena [14], known as Gap Junctions (GJ). The intestinal barrier impairment (‘‘leaky gut’’) is clearly considered one of the most important pathogenic mechanism in several chronic non-infectious diseases [15,16,17]. In CKD, the gut microbiota is significantly modified [18]: rising number of bacterial families possessing urease, uricase and indole and p-cresol-forming enzymes [19], lead to an increase of gut-derived uremic toxins production, such as IS [20,21,22]. Moreover, evidences indicate that in CKD conditions, the inflammatory state of the gastrointestinal tract in hemodialysis patients [23], as well as the disruption of the epithelial tight junctions throughout the gastrointestinal tract, in animal models of CKD, are associated with oxidative stress [24,25,26]. Previous studies have reported that IS is able to affect the intestinal tract [27], but the underling mechanism/s is not yet reported in intestinal epithelial cells (IECs), which play a pivotal role in intestinal system dysfunction. Here, we evaluated the effect of IS on oxidative and antioxidant stress response in vitro in IEC-6 cells, also studying the modification of cell monolayer. Moreover, the effect of CKD serum patients treated with AST-120 was also evaluated on IEC-6 cells.

## 2. Results

### 2.1. IS did not Affect Cellular Viability 

In order to evaluate the antiproliferative potential of IS on IEC-6, we evaluated IS effects (31.2–250 µM) on cellular viability. At all tested concentrations, IS did not produce any significant effect on IEC-6 proliferation (data not shown).

### 2.2. IS Enhanced ROS Release from IEC-6 Cells

**Figure 1:**
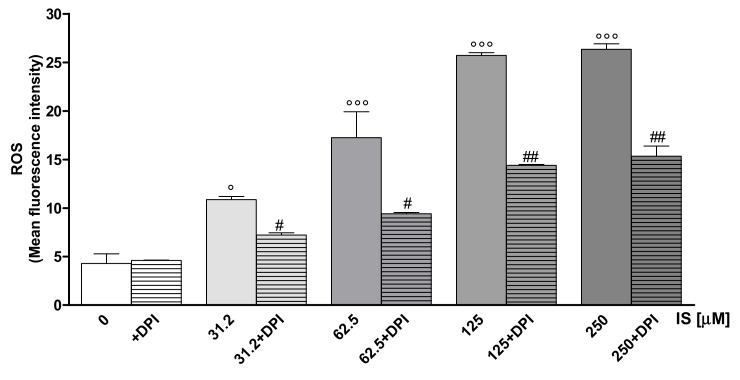
Effect of IS (31.2–250 μM), alone or in the presence of DPI, on ROS formation, evaluated by means of the probe 2′,7′ dichlorofluorescein-diacetate (H_2_DCF-DA) in IEC-6 cells. Fluorescence-activated cell sorting analysis (FACSscan; Becton Dickinson) was used to measure cellular fluorescence (*n* = 15). Data were elaborated with Cell Quest software. °°° and ° denote *p* < 0.001 and *p* < 0.05 vs. control; ## and # denote *p* < 0.01 and *p* < 0.05 vs. IS alone.

To investigate the effect of IS on oxidative stress in IEC-6 cells, we evaluated intracellular ROS release. Our results showed that IS significantly increased ROS production in IEC-6 cells (31.2–250 μM; *p* < 0.05 vs. control; Figure 1). In order to evaluate the role of NOXs in IS-induced ROS release in IEC-6 cells, in some experiments, intestinal cells were treated with Diphenyleneiodonium (DPI; 10 μM, a known antioxidant that inhibits flavoenzymes such as neutrophil NOX, was added 1 h before IS cellular treatment). When DPI was added to cells, ROS release was significantly inhibited at all tested concentrations (*p* < 0.05 vs. IS alone; Figure 1), thus indicating the involvement of NOX in IS-induced ROS production in IEC-6 cells.

### 2.3. IS Inhibits Nrf2 Nuclear Translocation and Modulated Heme Oxygenase-1 (HO-1), NAD(P)H Dehydrogenase (Quinone1) (NQO1) and Superoxide Dismutase (SOD) Expression in IEC-6 Cells

**Figure 2:**
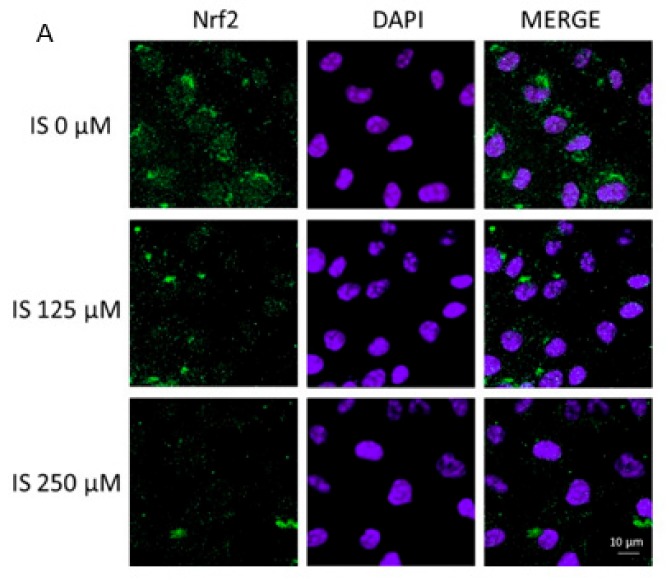

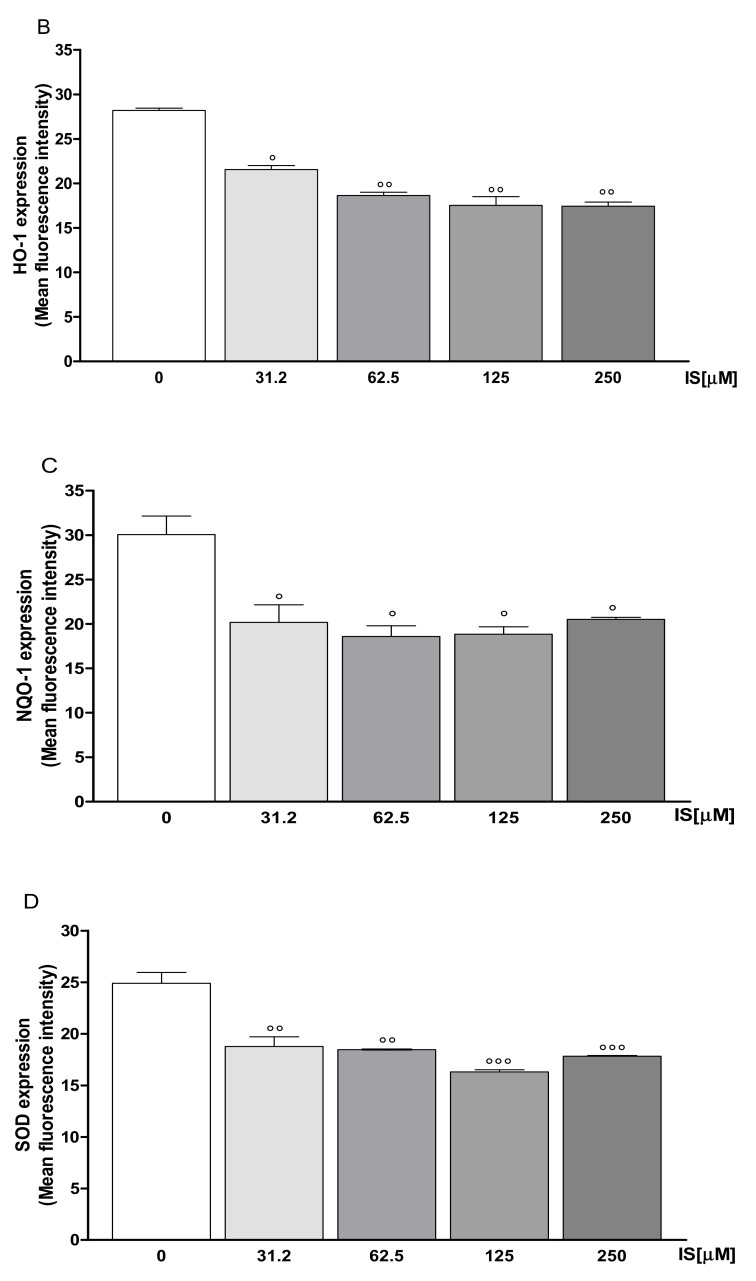
Effect of IS (125, 250 μM) on Nrf2 (Panel **A**) nuclear translocation in IEC-6 cells. Nrf2 translocation was observed using immunofluorescence confocal microscopy (*n* = 15). Scale bar, 10 µm. Blue fluorescence indicated the localization of nuclei (DAPI) and green indicated the localization of Nrf2. Effect of IS (31.2–250 μM) on HO-1 (Panel **B**), NQO1 (Panel **C**), SOD (Panel **D**) expression in IEC-6 cells. Values are expressed as mean fluorescence ± s.e.m. (*n* = 15). °°°, °° and ° denote respectively *p* < 0.001, *p* < 0.01 and *p* < 0.05 vs. control.

After being activated, Nuclear factor (erythroid-derived 2)-like 2 (Nrf2) moves from the cytosol into the nucleus and binds to specific sequences, influencing the expression of downstream genes aimed to regulate the anti-oxidant cellular response. In our experiments, Nrf2 was labelled with a green fluorescence probe, to track the effect of IS used at two average concentrations (125, 250 µM; added for 1 h), on its nuclear translocation. As shown in Figure 2A, IS (125, 250 µM) inhibited Nrf2 nuclear translocation with respect to the control cells. Moreover, we also assessed if IS (31.2–250 µM) added for 24 h to IEC-6 cells, influenced the expression of antioxidant enzymes, such as HO-1, NQO1 and SOD. Our results showed that HO-1, NQO1 and SOD expression were reduced by IS treatment (*p* < 0.05 vs. control; Figure 2B–D). 

### 2.4. Effect of IS on IEC-6 Cellular Migration

**Figure 3:**
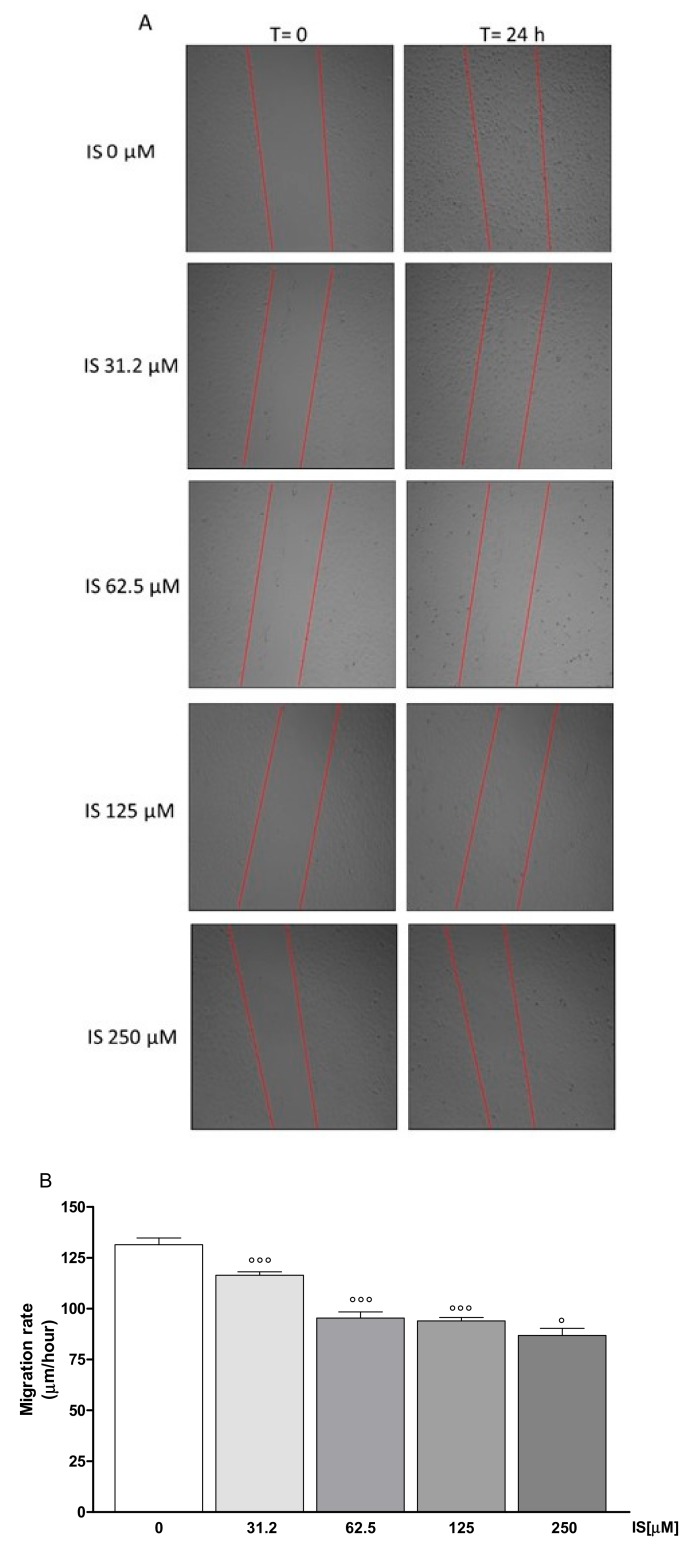

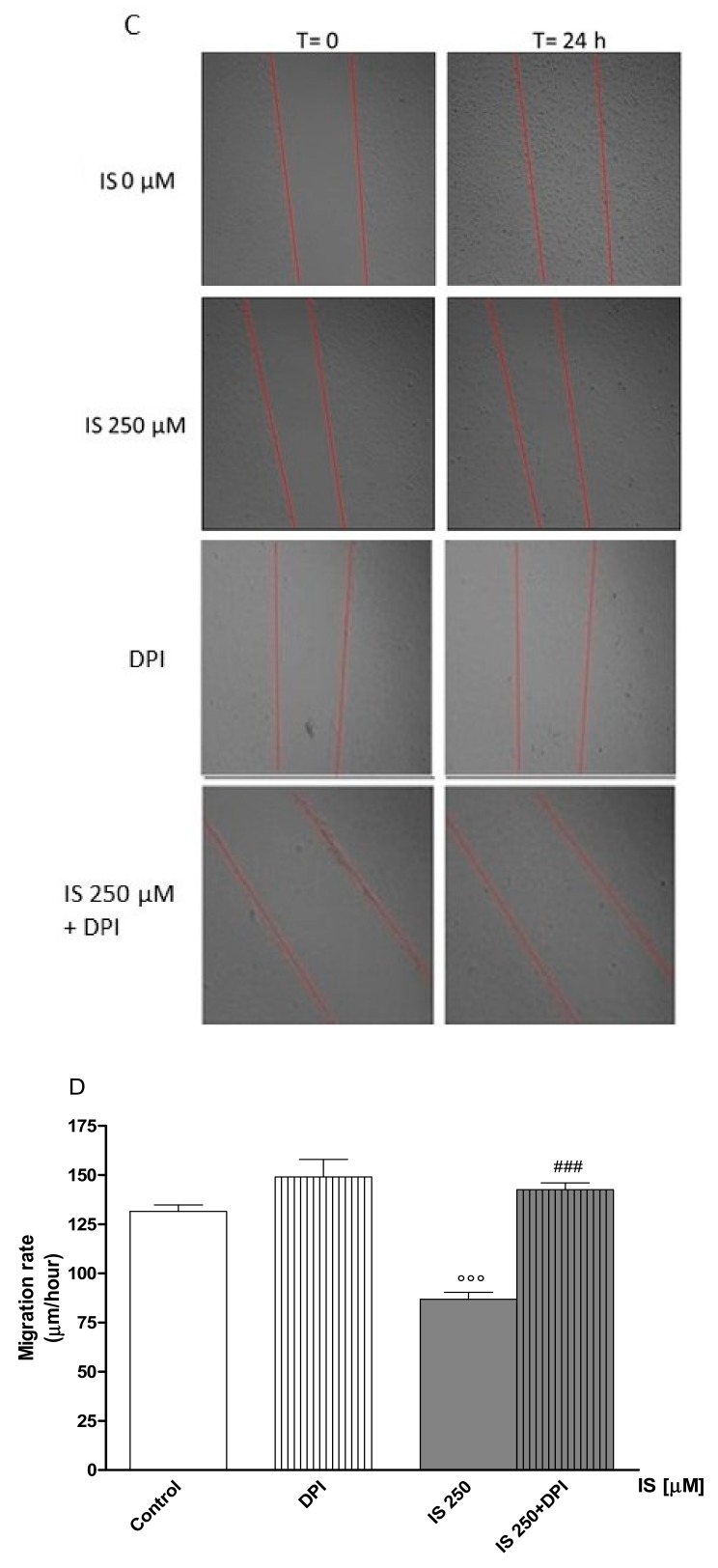
Pictures representing the wound repair induced by mechanical scratch in IEC-6 after 24 h from IS treatment (31.2–250 μM; **A**), and the quantitative analysis expressed as IEC-6 migration rate 24 h from the wound (**B**). Representative images of wound repair, in the presence of DPI and IS (250 μM; **C**) and its quantitative analysis expressed as IEC-6 migration rate after 24 h from the wound (**D**; *n* = 15). Values are expressed as migration rate. °°° and ° denote respectively *p* < 0.001 and *p* < 0.05 vs. control cells. ### denotes *p* < 0.001 vs. IS alone.

To assess the effect of IS on the reconstitution process at the intestinal level, we carried out a wound-healing assay on IS-treated IEC-6 monolayers. On complete confluence, a wound was created in IEC-6 monolayers by scraping and a time-lapse video microscopy was used to monitor cellular migration at the wound site for 24 h. Different cells were selected and their migration distances were measured at different time points. Figure 3A,B showed a significant decrease of the cellular migration speed of IEC-6 cells treated with graded IS concentrations (31.2–250 µM) compared to untreated cells (*p* < 0.05 vs. control; Figure 3A,B). In addition, to evaluate if IEC-6 cellular migration could be influenced by IS-induced ROS release, in some experiments, cells were treated with DPI (10 μM; 1 h before IS 250 µM cellular treatment). When DPI (10 µM) was added to IEC-6 cells, cellular migration speed was significantly enhanced, with respect to IS alone (*p* < 0.001 vs. IS alone; Figure 3C,D), thus indicating the contribution of IS-induced ROS in the observed cell migration reduction. 

### 2.5. IS Induced IEC-6 Cells Actin Cytoskeleton Rearrangement 

**Figure 4:**
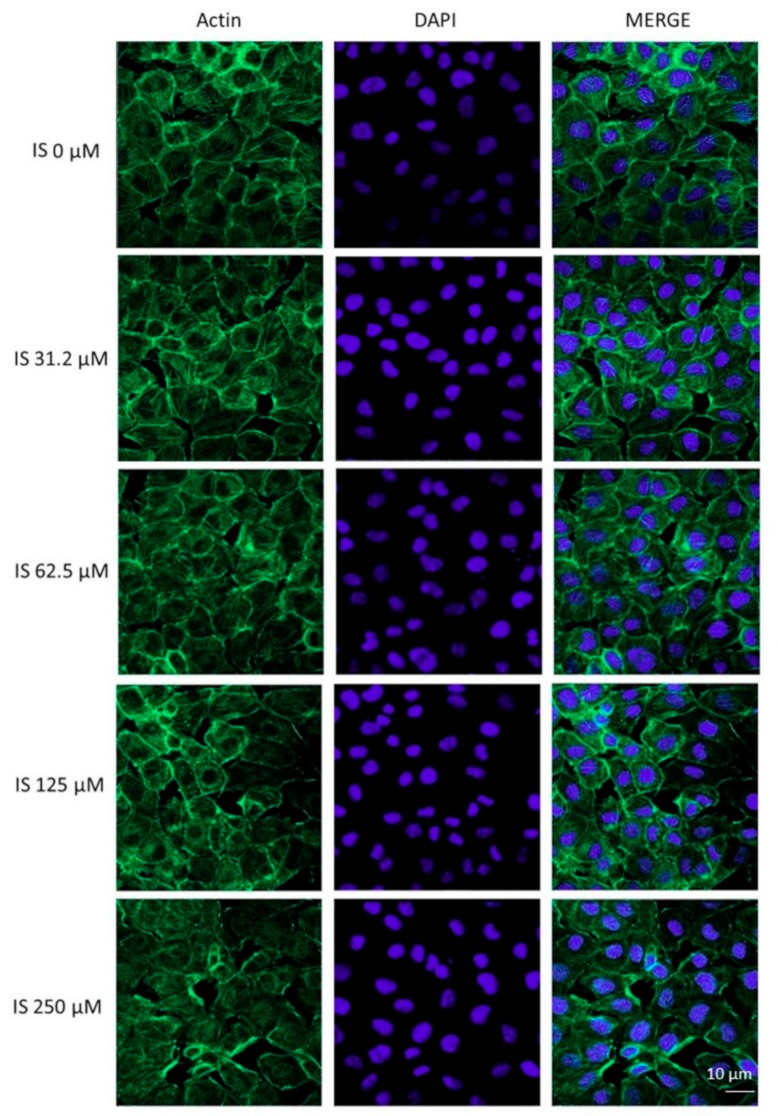
Effect of IS (31.2–250 μM) on the actin stress fibres assembly in IEC-6 cells. Immunofluorescence analysis was performed using immunofluorescence confocal microscopy (*n* = 15). Scale bar, 10 µm. Blue fluorescence indicated the localization of nuclei (DAPI) and green indicated the localization of actin stress fibres.

In order to examine some of the factors involved in IEC-6 epithelial wound repair inhibition induced by IS, we examined the effect of this uremic toxin (IS 31.2–250 µM) on the actin stress fibres’ assembly in IEC-6 cells. An intact actin cytoskeleton and actin fibres travel the cytosol in control cells (Figure 4). The treatment with IS, especially at the higher tested concentrations, caused a slight actin filament reorganisation to the cell subcortical compartment (Figure 4). 

### 2.6. Effect of IS on Connexin 43 Expression in IEC-6 Cells

**Figure 5:**
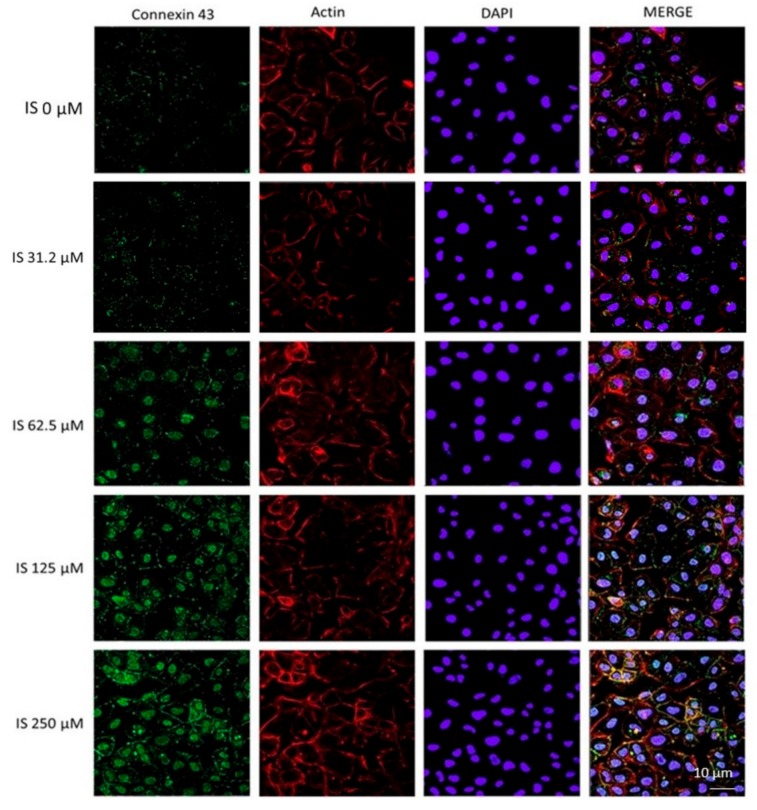
Effect of IS (31.2–250 μM) on connexin 43 expression in IEC-6 cells. Immunofluorescence analysis was performed by a immunofluorescence confocal microscopy (*n* = 15). Scale bar, 10 µm. Blue fluorescence indicated the localization of nuclei (DAPI), green indicated the localization of connexin 43 and red indicated the localization of actin fibres.

Considering that enterocytes migrate together, mucosal healing could require a communication of inter-enterocytes via gap junctions. Gap junctions are formed by transmembrane proteins, known as connexin and among them, connexin 43 is the most extensively investigated. By immunofluorescence analysis, we assessed that IS, especially at the concentration range between 31.2–250 µM, caused an increase and an alteration in connexin 43 distribution, from the membrane to the cytosol and cell nuclei (Figure 5).

### 2.7. IS Enhanced ROS Production in IEC-6 Cells: Effect of AST-120

**Figure 6:**
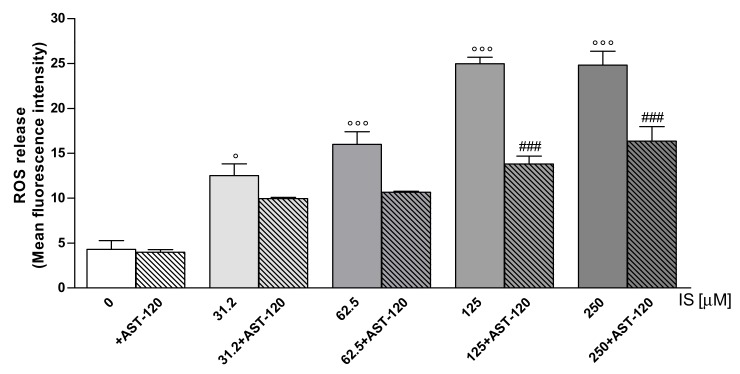
Effect on ROS formation of IS (31.2–250 μM) alone or in the presence of AST-120. Values are expressed as mean fluorescence intensity (*n* = 15). °°° and ° denote, respectively, *p* < 0.001 and *p* < 0.01 vs. control. ### denote *p* < 0.001 vs. IS alone.

The intracellular ROS release was evaluated in IEC-6 cells treated with IS (31.2–250 μM) alone or in the presence of an IS adsorbent: AST-120 (Figure 6). Our results showed that AST-120 treatment reduced ROS release from IEC-6 significantly only at the concentration range of 125 μM to 250 μM (*p* < 0.001 vs. IS alone; Figure 6).

### 2.8. IS in Sera of CKD Patients Increased ROS Production in IEC-6 Cells: Effect of AST-120

The IS effect on ROS release was evaluated in IEC-6 cells treated with sera derived from 11 subjects: sera from three human healthy people (H1, H2, H3), sera from four CKD patients (CKD1, CKD2, CKD3, CKD4) and sera from four CKD dialysed patients (HD1, HD2, HD3, HD4), characterized by different IS serum concentrations. IS levels in the sera subjects were calculated at baseline and at the end of the treatment with AST-120 by HPLC analysis [28], as shown in Table 1. 

**Figure 7:**
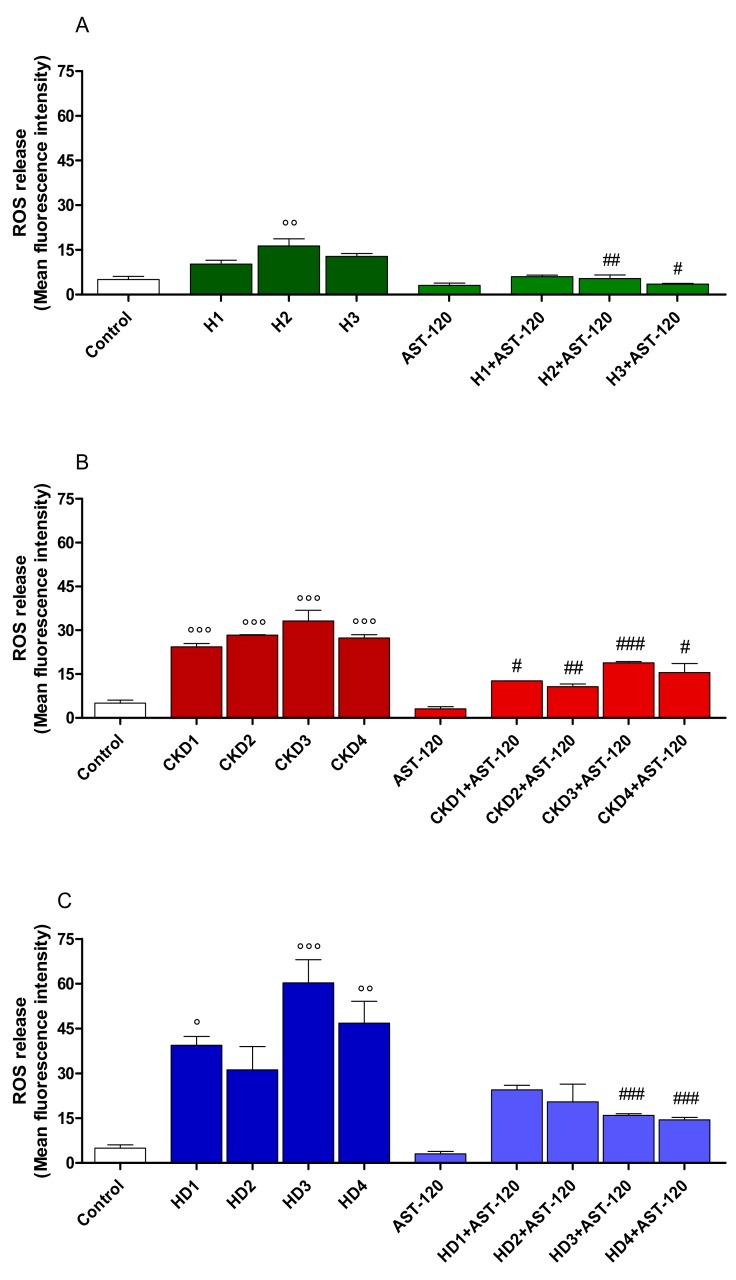

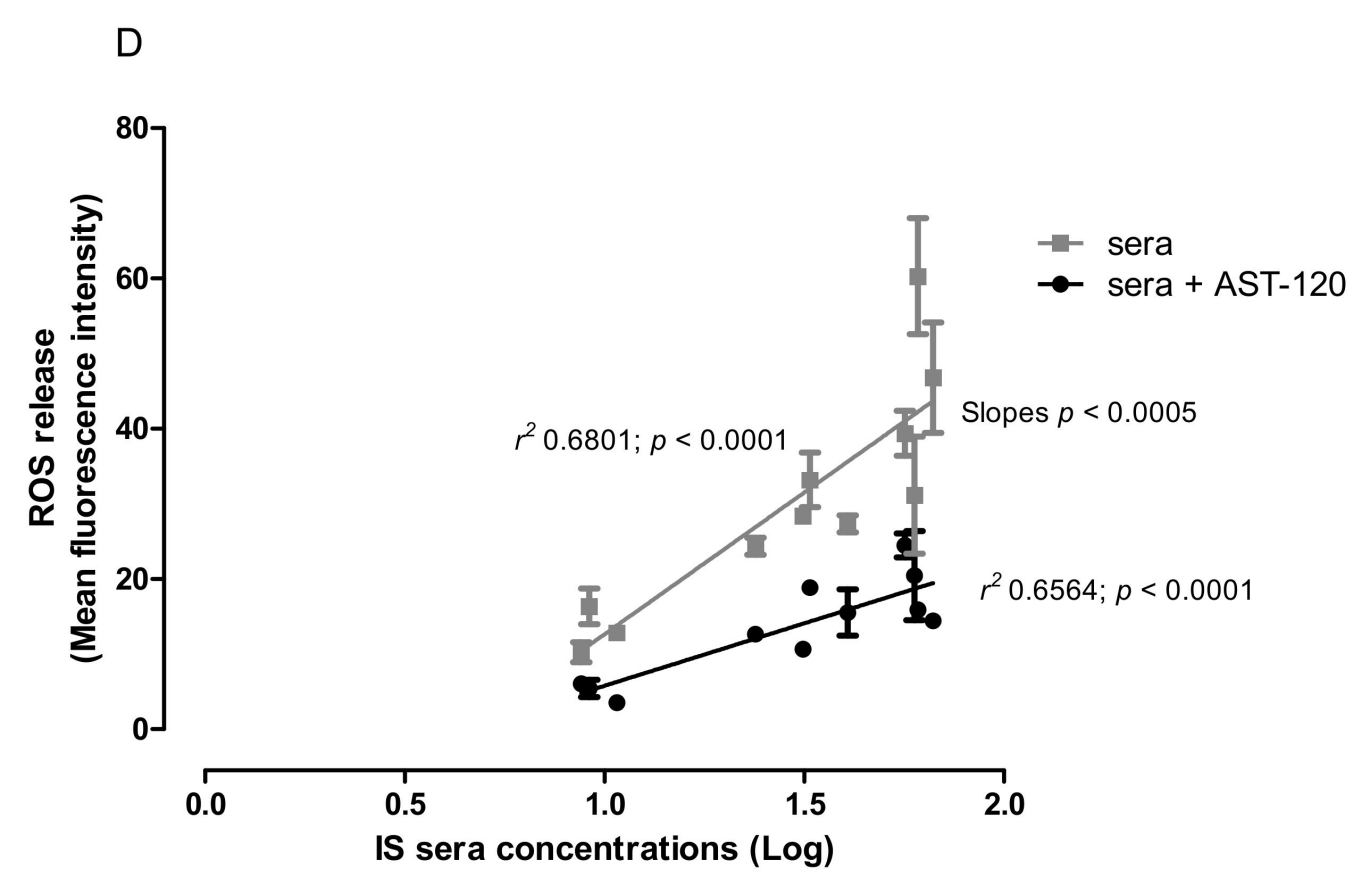
Effect on ROS formation of sera from eleven subjects: sera from three human healthy people (H1, H2, H3; Panel **A**), sera from four CKD patients (CKD1, CKD2, CKD3, CKD4; Panel **B**) and sera from four CKD dialysed patients (HD1, HD2, HD3, HD4; Panel **C**), alone or in the presence of AST-120 in IEC-6 cells (*n* = 15). Linear regression of ROS release by IEC-6 cells treated with sera alone or treated with AST-120 (Panel **D**; *n* = 3 measurement of each sample). Resulted indicated F = 10.9369, DFn = 1, DFd = 40, *p* < 0.0001 and the difference between the slopes are considered very significant (Panel **D**). Values are expressed as mean fluorescence intensity. °°°, °°, ° denote *p* < 0.001, *p* < 0.01 and *p* < 0.05 vs. control. ###, ##, # denote *p* < 0.001, *p* < 0.01 and *p* < 0.05 vs. serum alone.

Our data indicated that ROS release was weakly influenced in IEC-6 cells treated with sera deriving from human healthy people (H1-H3; *p* < 0.01 vs. control; Figure 7A), but was increased in the cells treated with sera derived from CKD patients (CKD1-CKD4; *p* < 0.001 vs. control; Figure 7B) and mostly in the cells treated with sera from CKD dialysed patients (HD1-HD4; *p* < 0.05 vs. control; Figure 7C). It is worth noting that there was a correlation between IS serum concentration, as determined by HPLC, and ROS production in IEC-6 cells. In fact, sera-induced ROS release in these cells was related to IS content in the sera of CKD patients, as indicated by r^2^ values and the difference between the slopes (Figure 7D). Moreover, AST-120 serum treatment significantly decreased ROS release from IEC-6 cells (Figure 7A–D), thus indicating IS contribution to the observed effects.

## 3. Discussion 

CKD is associated with various complications and CKD patients suffer from several organ dysfunctions related to disease progression. Intestine function is impaired during CKD, and a lot of interest is addressed at its potential role in other CKD aspects—oxidative stress and intestinal chronic inflammation [29]. The intestine takes part in uremic toxins production [29,30] and consequent accumulation in various organs and tissues [21,22]. Moreover, oxidative stress is enhanced with the worsening of renal function [31] and, therefore, with IS accumulation.

In this study, we reported that IS impairs IECs function, enhancing oxidative stress and affecting barrier repair with an ROS-dependent mechanism. Our results showed that IS induced a significant and concentration-related increase in ROS release from IEC-6 cells. Mechanistic studies, as revealed by DPI treatment, demonstrated that NOXs were implicated in IS-induced ROS release in our experimental model. Our results are in agreement both with previous studies from our research group, reporting that IS interfered with the same mechanism in macrophages [8] and glial cells [9] and with other reports on endothelial cells, vascular smooth muscle cells, and kidney cells.

In response to oxidative stress conditions, the organism keeps the balance between pro- and anti-oxidant systems [32]. Nrf2 is a transcription factor that plays a central role in regulating antioxidant and cytoprotective enzymes, including NQO1, HO-1 and SOD, which play a pivotal role in cellular defence against inflammation and oxidative stress [33,34]. Kelch like ECH-associated protein 1 (Keap1) binds Nrf2 in the cytoplasm, facilitating its ubiquitination and preventing translocation to the nucleus. Oxidative stress in CKD is characterized by an increase of Keap1 and impaired Nrf2 activity in the kidney and vascular tissues [35,36,37]. In our experimental model, Nrf2 nuclear activation was decreased by IS respect to the cellular control in IEC-6 cells. Our results are in accordance with previous research that shows that IS suppresses Nrf2 activation [38,39]. HO-1 is found in heme catabolism, the rate-limiting enzyme, and leads to the generation of biliverdin, carbon monoxide, and free iron and is a cytoprotective enzyme produced by Nrf-2 activation. Nrf-2 activation also induces NQO1 expression, an antioxidant flavoprotein that scavenges ROS. The enzyme SOD neutralizes O_2_^−•^ by transforming it into hydrogen peroxide (H_2_O_2_), and hydroxyl radical (HO•) [39,40,41]. Our results showed that HO-1, NQO1 and SOD expression were inhibited by IS, indicating a reduction of the anti-oxidant defense cellular systems, thus further contributing to oxidative stress. 

A main intestinal function is to form an intestinal barrier; the intactness of the IECs monolayer as well as the IECs’ function during the restitution process are of primary importance. Generally, the healing process is initiated in response to epithelial injury. Enterocytes migrate from healthy areas to the sites of mucosal lesion in order to fill up defects in the intestinal barrier. However, in various intestinal diseases, the intestinal healing is impaired, leading to persistent mucosal defects and potential consequences for the entire organism [42]. By means of a wound healing assay, we observed that IS decreased cellular migration speed in IEC-6, thus reducing the restitution process and affecting the intactness of the cellular monolayer. 

IEC-6 cells, when confluent, produce monolayers that resemble normal small intestinal cells and show an organized actin cytoskeleton [43,44]. A reorganisation of the actin cytoskeleton is fundamental during IECs’ restitution. In our experiments, we found that IS induced a marked reduction of actin stress fibres and a parallel increase of the cortical actin density, thus impairing IEC-6 movement and migration.

CKD is indirectly linked with damage to intestinal mucosa barrier function, which leads to several systemic consequences, such as endotoxemia presence in the absence of infection [24], bacterial translocation [45], and detection of intestinal bacteria DNA in the blood. In the enterocytes monolayer, it affects connections between closely arranged cells and juxtaposed IECs, gap junctions, modulated para- and intercellular transit of molecules. Gap junctional intercellular communication is an important, even if not completely understood, mechanism for tissue and cellular homeostasis that coordinates cell-cell passage of small metabolites and ions. Thus, gap junctional intercellular communication regulates cell proliferation, migration, and differentiation. Gap junction channels are formed by hexameric arrays of individual subunits known as connexions, and connexin 43 is the most expressed isoform [46,47]. A potential role for gap junctions in enterocyte migration regulation and thus in the healing process has been suggested [47]. Studies conducted in animal models of CKD, have observed a connection between the disruption of the epithelial gap junctions caused by changes in connexin 43 in the gastrointestinal tract and inflammation and oxidative stress [48], indicating a marked damage to the intestinal barrier. Our results showed that IS induced an increase and a different localization of connexin 43, being more present in the cytosol and nuclei than at the membrane level in IS-treated IEC-6 cell, leading to an impairment in gap junction communication and cellular migration ability. Moreover, the resulting nuclear connexin-43 is associated with a decrease in cellular proliferation [49], thus further supporting the effects of IS ability in interfering with the intactness of the IECs monolayer. 

Several uremic toxins have been proposed to significantly contribute to the “leaky gut” in CKD patients. Here, we also studied the effects of sera from CKD patients. In particular, we evaluated the intracellular ROS release induced by the sera alone or treated with AST-120 in IEC-6. AST-120, an intestinal adsorbent made of spherical carbon, consists of porous carbon particles that adsorb low-molecular-weight compounds such as IS, as well as *p*-cresyl sulfate and their precursors as indole and p-cresol, allowing them to be excreted in faeces before they can be absorbed into the bloodstream [50]. It is insoluble both in water and in organic solvents. If AST-120 is administered orally, it adsorbs IS, causing a reduction of IS serum and urinary concentrations [18]. We assessed the effect of sera from CKD patients, characterized by different IS concentrations. Our data indicated that sera from CKD patients, but mostly sera from CKD dialysed patients significantly, and in IS-concentration-related manner, increased ROS production in IEC-6 cells, with respect to the sera from healthy people. These results highlighted a direct relation between IS serum concentration and the ability to induce ROS production in IEC-6. Moreover, we outlined that ROS release was significantly reduced in the presence of AST-120, due to its absorption of some uremic toxins. These evidences agree with a previous study reporting the AST-120 capability to improve intestinal function in CKD and supporting, in particular its antioxidant effects during CKD [51,52]. In fact, clinical trials have demonstrated that AST-120 reduces serum IS levels in a dose-dependent manner, thus ameliorating renal function decline, improving at least some symptoms of uremia [53], and reducing oxidative stress [54,55,56]. 

Our results indicated that among the various uremic toxins accumulating in CKD patients, IS significantly contributes to IECs impairment, thus, highlighting IS as a potential pharmacological target in CKD.

## 4. Materials and Methods

### 4.1. Reagents

All compounds and reagents were purchased from Sigma Chemicals Company (Sigma, Milan, Italy), unless stated otherwise.

### 4.2. Cell Culture 

The IEC-6 cell line (CRL-1592) was purchased from the American Type Culture Collection (ATCC, Rockville USA/Maryland). IEC-6 cells are a non-tumorigenic cell line, derived from normal rat intestinal crypt cells [56]. This cell line was cultured with Dulbecco’s modified Eagle’s medium (DMEM, 4 g/L glucose) containing 2 mM L-glutamine, 10% (*v*/*v*) fetal bovine serum (FBS), 0.1 unit/mL bovine insulin and 1.5 g/L NaHCO_3_. For the experiments, IEC-6 cells were used between the 16th–19th passages.

### 4.3. IS Experiments on IEC-6 Cells

#### 4.3.1. Cellular Treatment 

The IEC-6 cells were plated and, after adhesion (24 h), were treated with IS (31.2–250 μM) for different experimental times, as highlighted below. In some experiments, in order to estimate the possible involvement of NOXs, both in ROS release and in wound healing assay, DPI (10 μM), was added 1 h before IS cellular treatment.

#### 4.3.2. Cellular Viability

IEC-6 cells (2 × 10^4^ cells/well; 96-well plates) were plated and allowed to adhere. IS (31.2–250 μM) was then added to the cells for 24 h. Then, 25 µL of MTT (5 mg/mL) were added for 3 h. Cells were then lysed and the dark blue crystals solubilised with 100 µL of a solution containing 50% (*v*:*v*), N-dimethylformamide, 20% (*w*:*v*) SDS with an adjusted pH of 4.5. A microplate spectrophotometer (Titertek Multiskan MCC/340-DASIT), equipped with a 620 nm filter, was used to measure the optical density (OD) of each well. Cells viability was calculated as: % dead cells = 100 − [(OD treated/OD control) × 100], as reported [57].

#### 4.3.3. Intracellular ROS Evaluation

Intracellular ROS levels were measured by the fluorescent probe 2′,7′-dichlorofluorescin-diacetate (H_2_DCF-DA) [58]. IEC-6 cells were plated (8 × 10^4^ cells/well; 24-well plates) and treated with IS (31.2–250 μM) for 24 h. In some experiments, DPI (10 μM) was added 1 h before IS treatment. Cell fluorescence was evaluated, after 15 min, by a FACSscan (Becton Dickinson, Milan, Italy) and data were elaborated with Cell Quest software. 

#### 4.3.4. Immunofluorescence Analysis by Confocal Microscopy of Nuclear Factor (Erythroid-Derived 2)-like 2 (Nrf2) Translocation and Connexin 43 Expression 

For immunofluorescence analysis, IEC-6 cells (2 × 10^5^/well; 12 well plate) were treated with IS (125, 250 μM) for 1 h, to evaluate Nrf2 nuclear translocation, or for 24 h, to evaluate connexin 43 expression. IEC-6 cells were fixed with 4% paraformaldehyde in PBS for 15 min and then permeabilized with phosphate-buffered saline (PBS), containing 0.1% saponin for 15 min. A blocking, for 1 h, with bovine serum albumin (BSA) and PBS was then performed, cells were then incubated with anti-Nrf2 (sc-722 Santa Cruz Biotechnology, Santa Cruz, CA, USA) or with anti-connexin 43 antibody (sc-9059 Santa Cruz Biotechnology) at 37 °C. After 1 h, the slides were washed with PBS and fluorescein-conjugated (FITC) secondary antibody was added, for 1 h. For the evaluation of connexin 43 expression, we incubated the slides with Phalloidin-tetramethyl rhodamine B isothiocyanate-conjugated anti-F-actin (1 mg/mL in PBS for 30 min). 4′,6-diamidin-2-phenylindol (DAPI) was used to stain the nuclei for both evaluations. Finally, coverslips were mounted using mounting medium and fluorescent images were taken under a Laser Confocal Microscope (Leica TCS SP5), as reported [59].

#### 4.3.5. Evaluation by Cytofluorimetry of Heme Oxygenase-1 (HO-1), NAD(P)H Dehydrogenase (Quinone1) (NQO1) and Superoxide Dismutase (SOD).

To evaluate HO-1, NQO1 and SOD expression, IEC-6 cells (2 × 10^4^ cells/well; 96-well plates) were treated with IS (31.2–250 μM) for 24 h.

Thereafter, the cells were collected, washed with PBS, and incubated with Fixing Solution for 20 min at 4 °C and successively for 30 min at 4 °C with Fix Perm Solution. Anti-HO-1 (sc-10789, Santa Cruz Biotechnology) or anti-NQO1 (sc-376023, Santa Cruz Biotechnology) or anti-SOD (sc-30080, Santa Cruz Biotechnology) antibody were subsequently added. The secondary antibody was added, for 30 min, in Fix Solution, and cell fluorescence was evaluated by FACSscan (Becton Dickinson) and elaborated with Cell Quest software, as previously reported [60].

#### 4.3.6. Wound-Healing Assay

In order to evaluate cellular migration in IS-treated IEC-6 cells, a wound-healing assay was performed, as previously reported [61]. IEC-6 cells (1 × 10^5^ cells/well, 24-well plates) were allowed to adhere for 24 h. A mechanical wound was induced at the centre of the IEC-6 monolayer by scraping cells with a sterile plastic p200 pipette tip. Cells were then washed with PBS and treated with IS (31.2–250 μM) for 24 h. One hour before IS treatment, DPI (10 μM) was added, in some experiments. The wounded IEC-6 cells were then placed in a humidified and equilibrated (5% *v*/*v* CO_2_) incubation chamber of an Integrated Live Cell Workstation Leica AF-6000 LX at 37 °C for 24 h. A 10X phase contrast objective was used in order to record cell movements, with a frequency of acquisition of 10 min. To determine the migration rate of individual cell, we considered the distances covered from the initial time to the selected time-points (bar of distance tool, Leica AF software, 2.3.5 build 5379, Leica, Wetzlar, Germany). For each wound, at least three different positions were registered and, to measure the migration distances, for each position, at least 10 different cells were randomly selected. GraphPad Prism 4 software (GraphPad, San Diego, CA, USA) was used to perform the statistical analyses.

#### 4.3.7. Immunofluorescence Assay for Cytoskeleton Analysis by Confocal Microscopy 

To evaluate IS effects on cellular cytoskeleton, IEC-6 cells were seeded on coverslips in 12 well plate (2 × 10^5^/well) and treated with IS (31.2–250 μM) for 24 h. Cells were fixed with 4% paraformaldehyde in PBS for 15 min and permeabilized with 0.1% saponin in PBS for 15 min. Slides were then incubated with FITC-conjugated anti-F-actin (Phalloidin-FITC, Sigma, Milan, Italy) at the concentration of 1 mg/mL in PBS for 30 min. The slides were then washed three times with PBS and DAPI was used for counterstaining of nuclei. Coverslips were finally mounted in mounting medium and fluorescent images were taken under the Laser Confocal Microscope (Leica TCS SP5, Wetzlar, Germany), as previously described [59].

### 4.4. CKD Serum Patients Experiments

#### 4.4.1. CKD Serum Patients Studies on IEC-6 Cells

This is a post hoc analysis of a preceding study [62,63]. The study was approved by the Ethics Committee of the Campania Nord and carried out in accordance with the current version of the Declaration of Helsinki. We analysed the sera of 11 subjects: three healthy people (H), four CKD patients (CKD) and four CKD dialysed patients (HD). IS sera levels were measured at baseline and at the end of the treatment with AST-120. This was demonstrated by HPLC analysis, carried out according to methods proposed by Zhu [28], as previously reported [10,64]. AST-120 was provided by Kureha Corporation.

#### 4.4.2. IEC-6 Cells Treatment with Sera from CKD Patients

After adhesion, IEC-6 cells were treated with sera from 11 subjects: three healthy people (H), four CKD patients (CKD) and four CKD dialysed patients (HD), (sera dilution 1:50 in culture media) for 24 h. In other experiments, before cellular treatment, the serum samples were incubated at 37 °C, for 3 h, with 1 g/dl AST-120, as previously reported. Thereafter, AST-120 bound to IS was settled as a pellet and the supernatants were filtered and administrated to the IEC-6 cells (sera dilution 1:50 in the culture media) for 24 h. ROS release was then evaluated.

### 4.5. Data Analysis

Data are reported as mean ± standard error mean (s.e.m.) values, of at least five independent experiments each in triplicate. Statistical analysis was carried out by analysis of the variance test and Bonferroni’s test was used for multiple comparisons. A *p*-value less than 0.05 was considered significant.

## 5. Conclusions

The impaired intestinal function is well recognized as a key factor in different pathologies such as in CKD-associated complications. The uremic toxin IS significantly enhanced oxidative stress in IECs and the IS-induced intestinal barrier damage could be addressed to its pro-oxidant effect. These results indicate that, among toxins present in sera from CKD patients, IS significantly and in a concentration-related manner contributed to oxidative stress in IEC-6; it was also involved in the inflammatory process, tightly linked to intestinal damage caused by uremia. Thus, the control of IS sera concentration in CKD patients (e.g., with AST-120) could be of significant help to rapidly decrease oxidative stress in IECs.

## Figures and Tables

**Table 1:** IS concentrations in sera from 11 subjects: sera from three human healthy people (H1, H2, H3), sera from four CKD patients (CKD1, CKD2, CKD3, CKD4) and sera from four CKD dialysed patients (HD1, HD2, HD3, HD4); with or without AST-120 treatment. *** denotes *p* < 0.001 vs. serum alone.

	IS (µM)	IS (µM) + AST-120
H 1	8.75 ± 0.1	8.15 ± 0.5
H 2	9.15 ± 0.2	8.75 ± 0.3
H 3	10.74 ± 0.6	7.56 ± 0.3
CKD 1	23.87 ± 0.2	21.48 ± 0.5
CKD 2	31.43 ± 0.3	29.04 ± 0.2
CKD 3	32.66 ± 0.6	27.85 ± 0.6
CKD 4	40.58 ± 1.1	35.81 ± 0.5
HD 1	56.50 ± 1.2	38.20 ± 0.9 ***
HD 2	59.58 ± 0.8	40.98 ± 1.1 ***
HD3	60.88 ± 1.5	43.77 ± 0.8 ***
HD 4	66.45 ± 0.5	48.15 ± 2.3 ***
